# Surface characterization data for tethered polyacrylic acid layers synthesized on polysulfone surfaces

**DOI:** 10.1016/j.dib.2019.103747

**Published:** 2019-03-07

**Authors:** Soomin Kim, Kari J. Moses, Shivani Sharma, Muhammad Bilal, Yoram Cohen

**Affiliations:** a Department of Chemical and Biomolecular Engineering, University of California, Los Angeles, CA 90095, USA; b California NanoSystems Institute, University of California, Los Angeles, CA 90095, USA; c Department of Chemistry and Biochemistry, University of California, Los Angeles, CA 90095, USA

## Abstract

The data presented are supplementary to an article [Kim et al., 2019] on synthesis and surface characterization of tethered polyacrylic acid (PAA) layers on polysulfone (PSf) film/membrane surfaces via atmospheric pressure plasma-induced graft polymerization (APPIGP). Data on surface characterization of the synthesized tethered PAA layers includes: AFM topographic surface images and height distributions of surface features, dry layer thickness, chain rupture length distributions determined via AFM based force spectroscopy (AFM-FS), in addition to measurements of water contact angles. Fouling propensity data for ultrafiltration of alginic acid as a model foulant are also provided for native and PAA grafted PSf ultrafiltration (UF) membranes.

**Table:** Specifications table

Subject area	*Material science*
More specific subject area	*Graft polymerization, tethered polymer layers, surface hydrophilicity, surface fouling resistance*
Type of data	*Table, image (FIB-SEM, AFM), figure*
How data were acquired	*XPS (Kratos Axis Ultra DLD)* *FIB-SEM (FEI Nova 600 NanoLab DualBeam*^*TM*^*-SEM/FIB*) *AFM (surface topography: Bruker Dimension Icon SPM with a NanoScope V controller; AFM force spectroscopy: Bruker MultiMode 8-HR SPM with a PicoForce Spectroscopy Control Module)*
Data format	*Raw and analyzed*
Experimental factors	*Tethered polyacrylic acid chains were graft polymerized onto polysulfone surfaces post atmospheric pressure plasma surface activation.*
Experimental features	*Surface characterization was performed for tethered polyacrylic acid layers synthesized onto polysulfone surfaces.*
Data source location	*University of California, Los Angeles (UCLA), Los Angeles, California, USA.*
Data accessibility	*Data are within this article.*
Related research article	*S. Kim, Y. Cohen, K.J. Moses, S. Sharma, M. Bilal, Polysulfone surface nano-structured with tethered polyacrylic acid, Applied Surface Science, 470 (2019) 411–422.*

**Table:** 

**Value of the data** • *The data provide quantitative information regarding the characteristics (surface topography, wettability, chain length distributions) of tethered polyacrylic acid layer under various solvents. Such surfaces have potential applications as fouling resistant surfaces including in membrane filtration and in biomedical applications.* • *The provided detailed AFM-FS data should be useful to researchers who may use the technique to evaluate surface tethered polymer chain lengths under different solvent conditions, as well as evaluate various algorithms for exploring AFM-FS data.* • *Membrane fouling data should be useful to researchers who may conduct membrane fouling tests under similar experimental conditions.*

## 1 Data

**Fig. 1:**
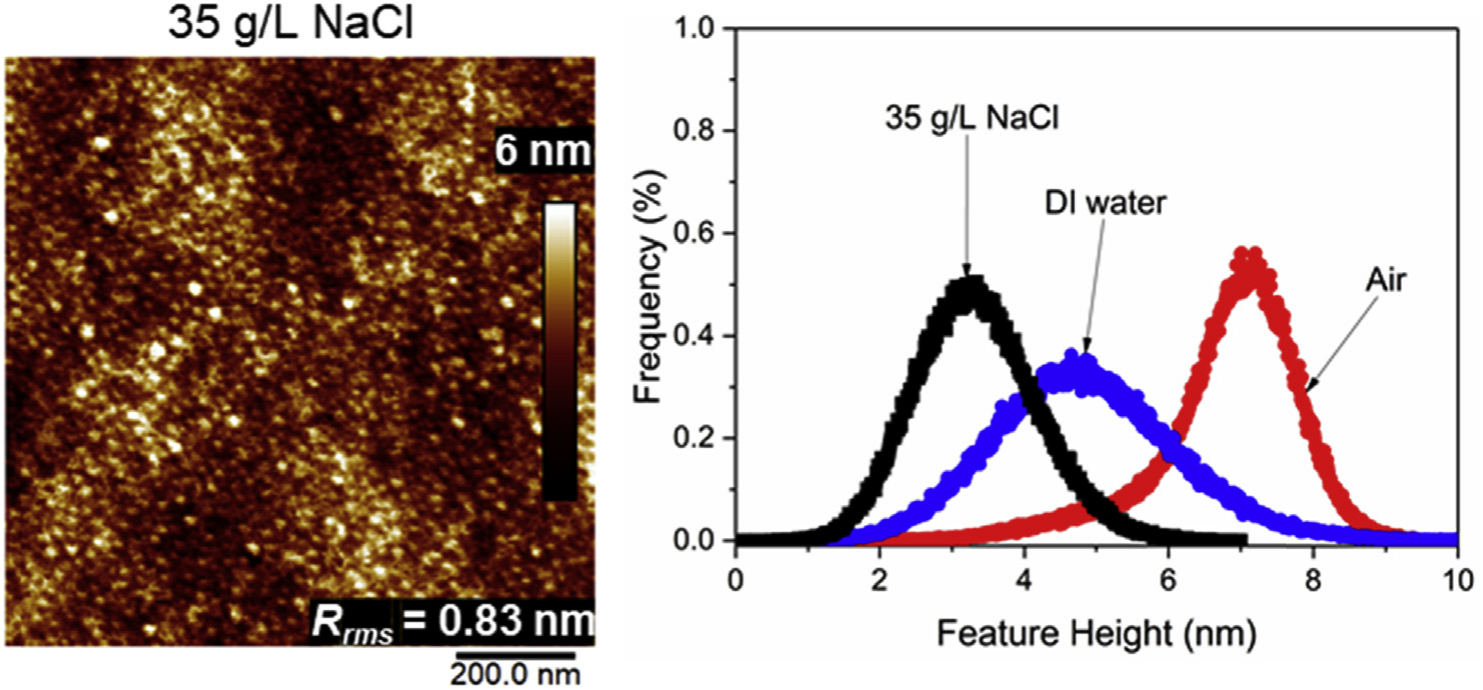
AFM 2-D image obtained in 35 g/L NaCl solution (left) and the corresponding feature height distribution (FHD) (right) for the PAA-PSf-Si (the tethered PAA layer synthesized at [M]_0_ = 20 vol% for 2 h following surface activation with He/O_2_ APP) (plotted along with the FHD for the surface as determined in DI water and air). The feature heights are scaled to the lowest height determined by the AFM tip and *R*_*rm*_ is root-mean-square surface roughness.

**Fig. 2:**
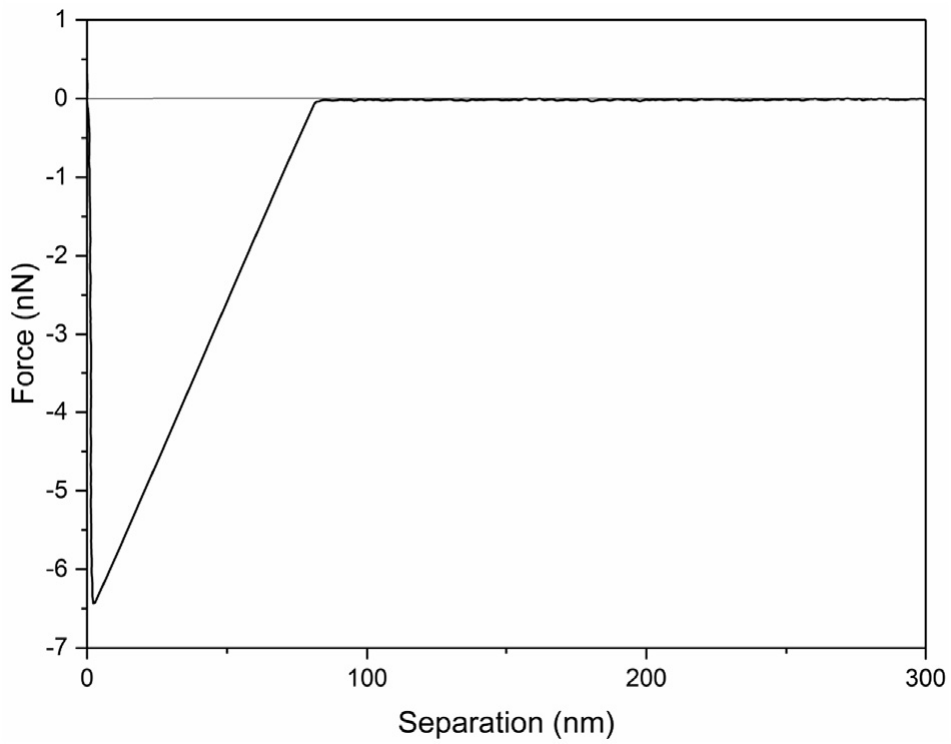
A typical retraction force curve for the native PSf-Si surface in DI water.

**Fig. 3:**
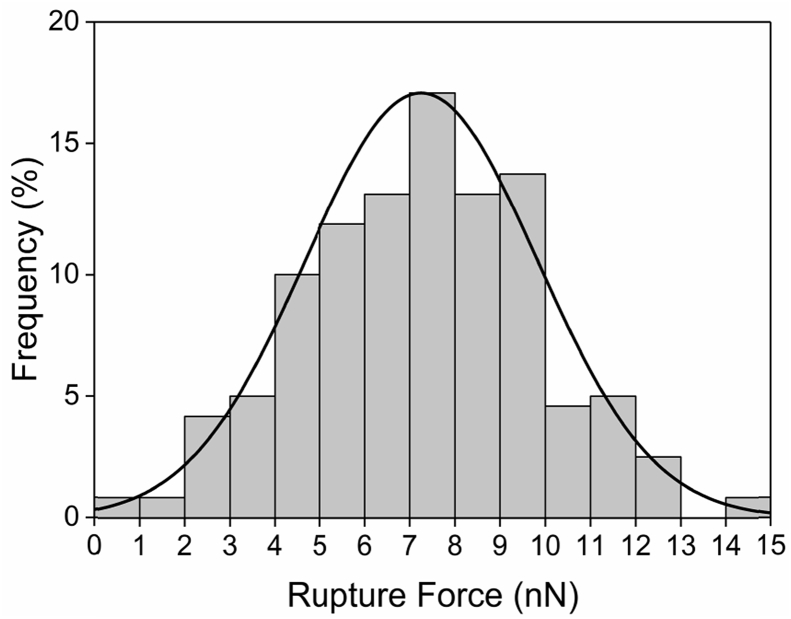
AFM-FS determined rupture force distribution for the native PSf-Si surface in DI water.

**Fig. 4:**
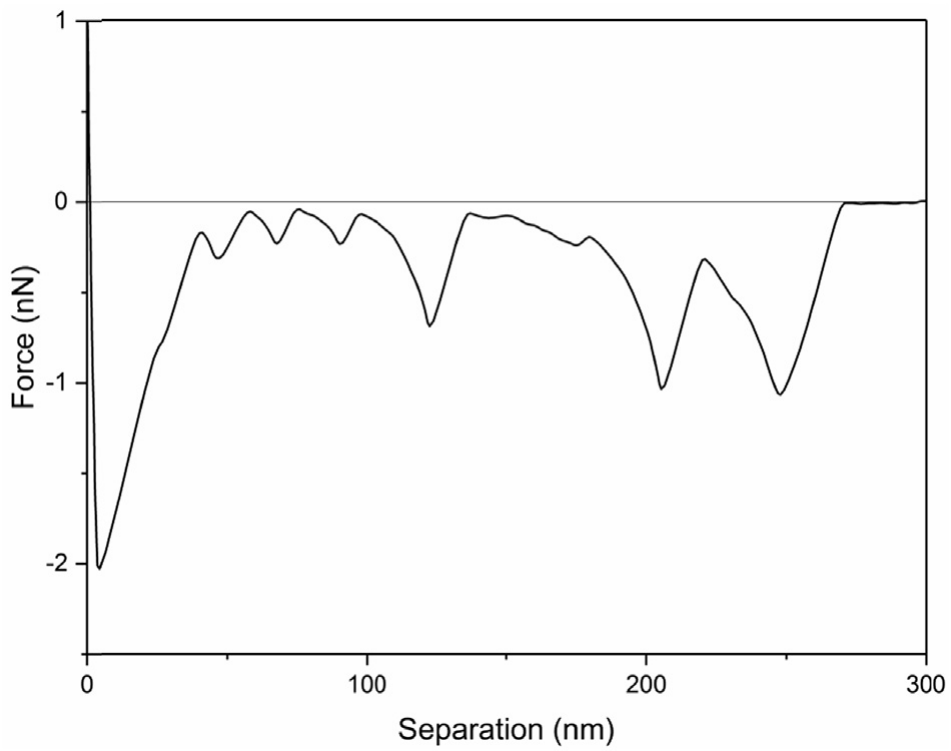
A typical retraction force curve for PSf with the tethered PAA layer (PAA-PSf-Si) in DI water. The tethered PAA layer (synthesized onto PSf activated with He/O_2_ plasma) was synthesized at [M]_0_ = 20 vol% for 2 h.

**Fig. 5:**
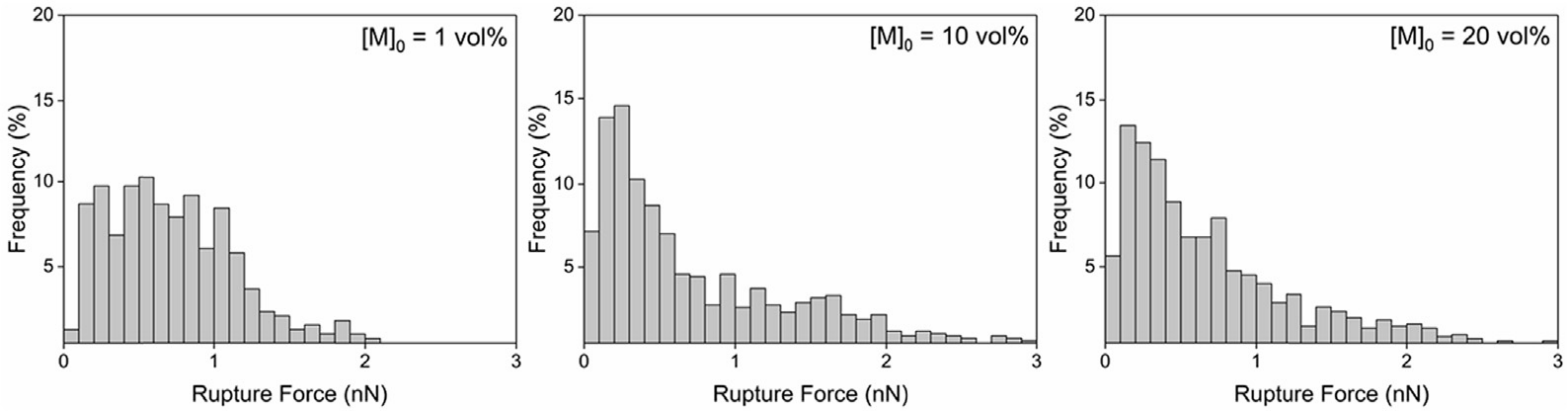
AFM-FS determined rupture force distributions (evaluated under DI water) for PAA chains synthesized at [M]_0_ of 1 vol%, 10 vol%, and 20 vol% for 2 h (Note: the PSf surfaces were activated with He/O_2_ plasma; data include the surface adhesion peaks (i.e., the first rupture event in the retraction profiles, *L*_*R*_ < 10 nm)).

**Fig. 6:**
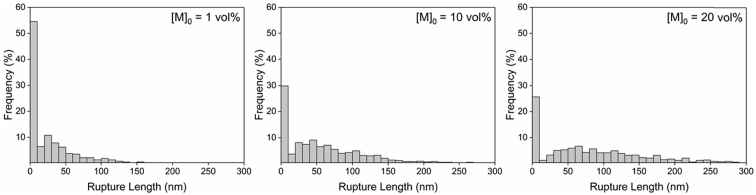
AFM-FS determined rupture length distributions (evaluated under DI water) for PAA chains synthesized at [M]_0_ of 1 vol%, 10 vol%, and 20 vol% for 2 h (Note: the PSf surfaces were activated with He/O_2_ plasma; data include the surface adhesion peaks (i.e., the first rupture event in the retraction profiles, *L*_*R*_ < 10 nm)).

**Fig. 7:**
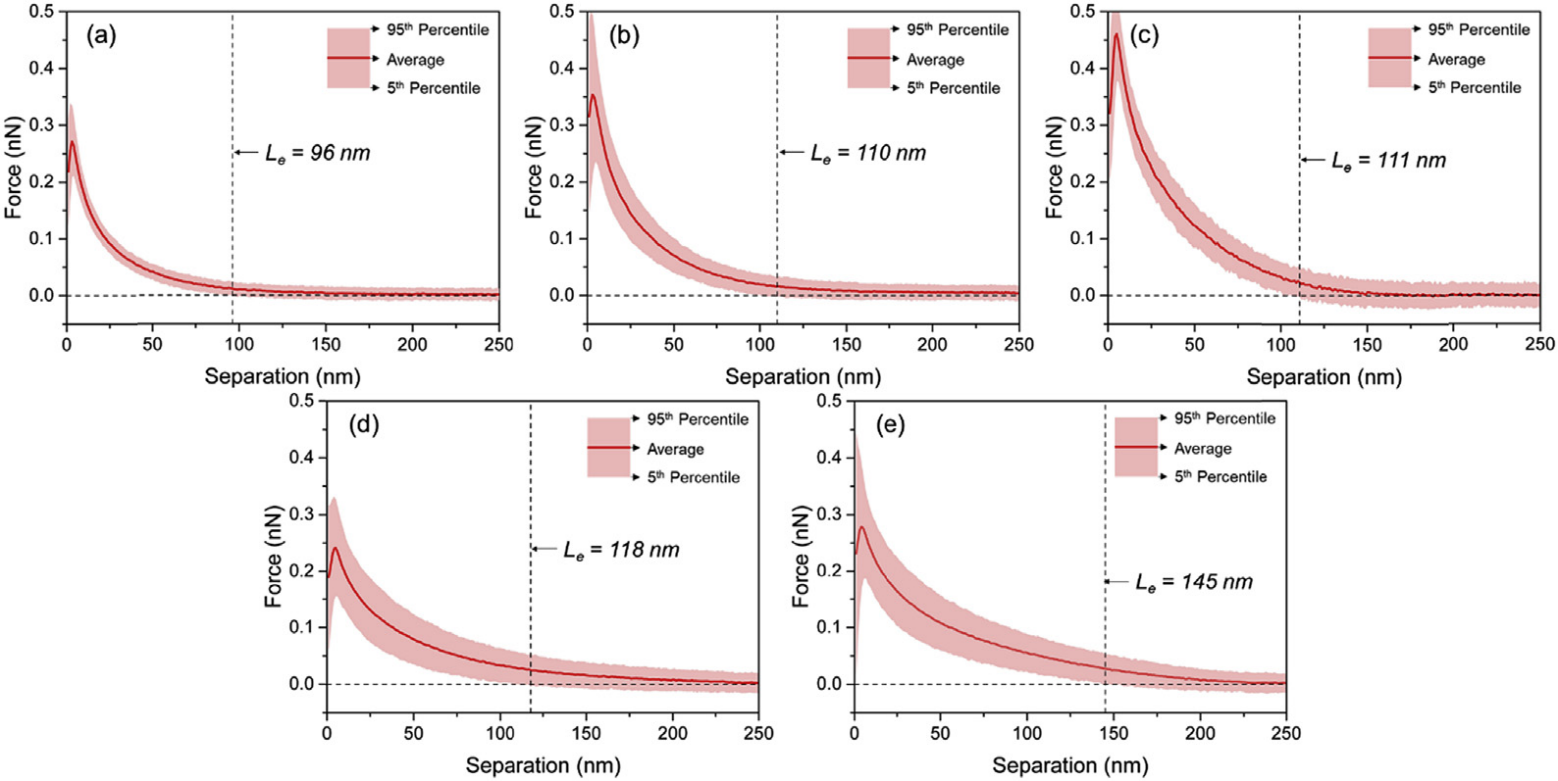
Equilibrium thickness (*L*_*e*_) in DI water estimated for PSf with the tethered PAA layers (PAA-PSf-Si) synthesized at [M]_0_: (a) 1 vol%, (b) 5 vol%, (c) 10 vol%, (d) 15 vol%, and (e) 20 vol% for a reaction period of 2 h (Note: PSf surface was activated with He/O_2_ plasma).

**Fig. 8:**
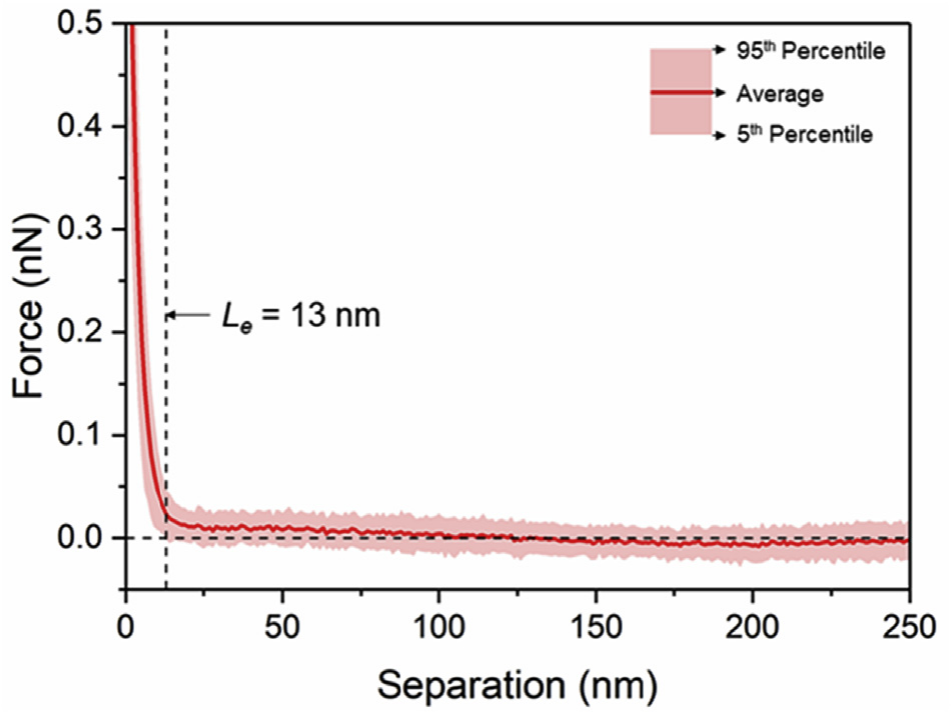
Equilibrium thickness (*L*_*e*_) in saline water (35 g/L NaCl) estimated for PSf with the tethered PAA layer (PAA-PSf-Si) synthesized at [M]_0_ = 20 vol% for a reaction period of 2 h (Note: PSf surface was activated with He/O_2_ plasma).

**Fig. 9:**
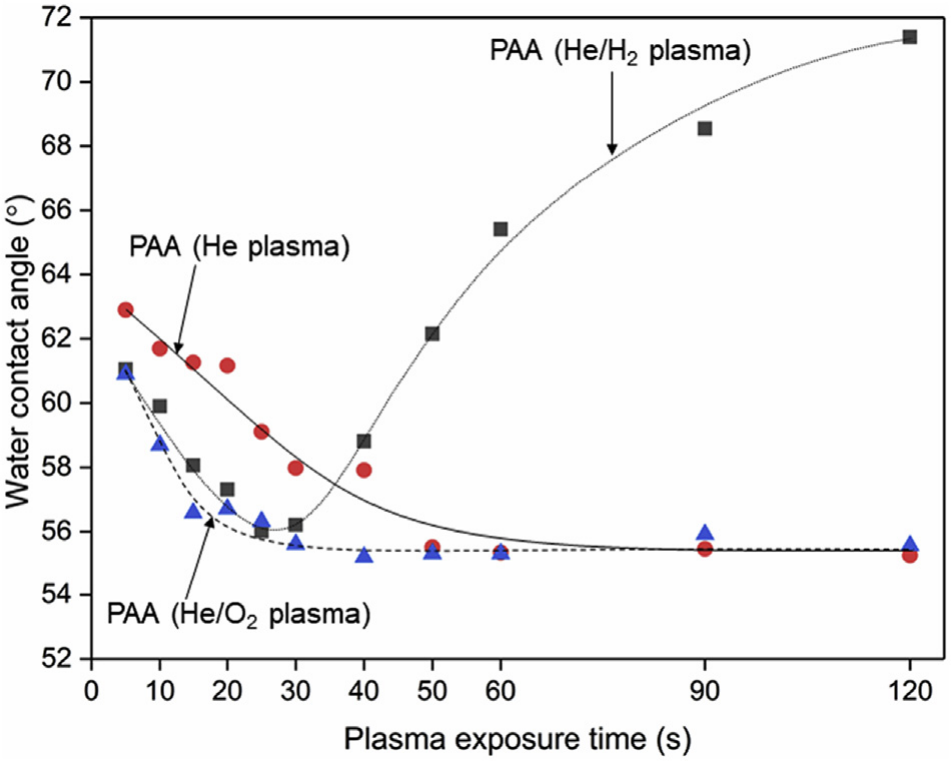
Sessile drop water contact angles for PAA-PSf-Si surfaces (graft polymerized at [M]_0_ = 10 vol% for 1 h) following PSf surface activation with He/H_2_, He, and He/O_2_ APP at various plasma exposure times (5–120 s). Water contact angles increased by ∼7% after storage in air for ≥5 days for the tethered PAA layers regardless of the type of APP used for surface activation prior to graft polymerization.

**Fig. 10:**
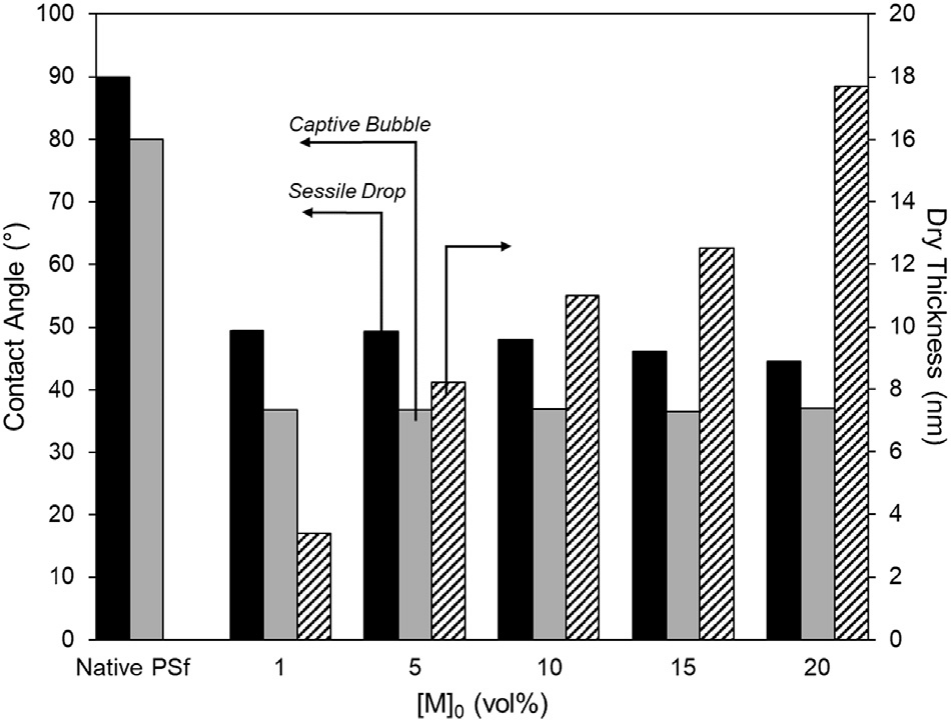
Sessile drop and captive bubble contact angles for the native PSf-Si and for PSf with the tethered PAA layer (PAA-PSf-Si) synthesized at [M]_0_ = 1–20 vol% for 2 h post-PSf surface activation with He/O_2_ plasma. Dry thickness is also plotted for the tethered PAA layers.

**Fig. 11:**
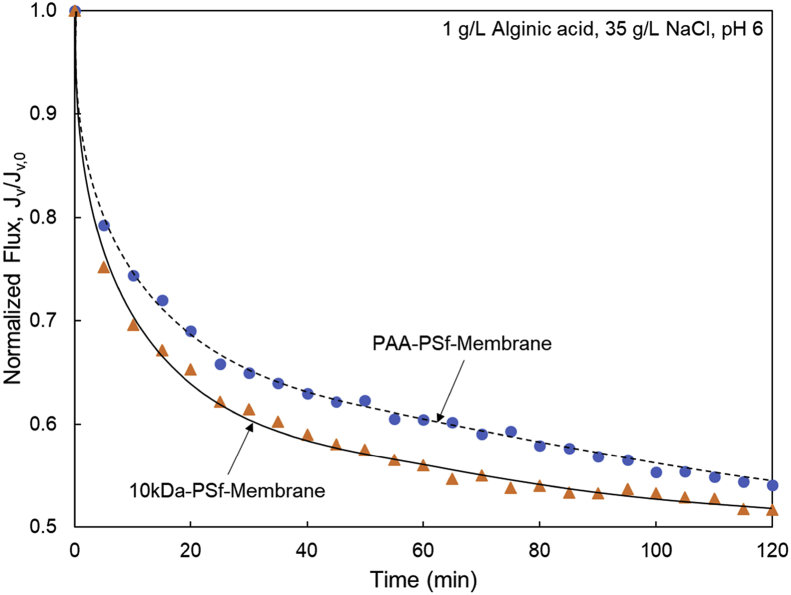
Permeate flux decline during filtration of saline alginic acid solution at pH 6 with (a) PAA-PSf-Membrane (synthesized at [M]_0_ = 20 vol% for 1 h post PSf surface activation with He/O_2_ plasma) and (b) 10kDa-PSf-Membrane.

**Fig. 12:**
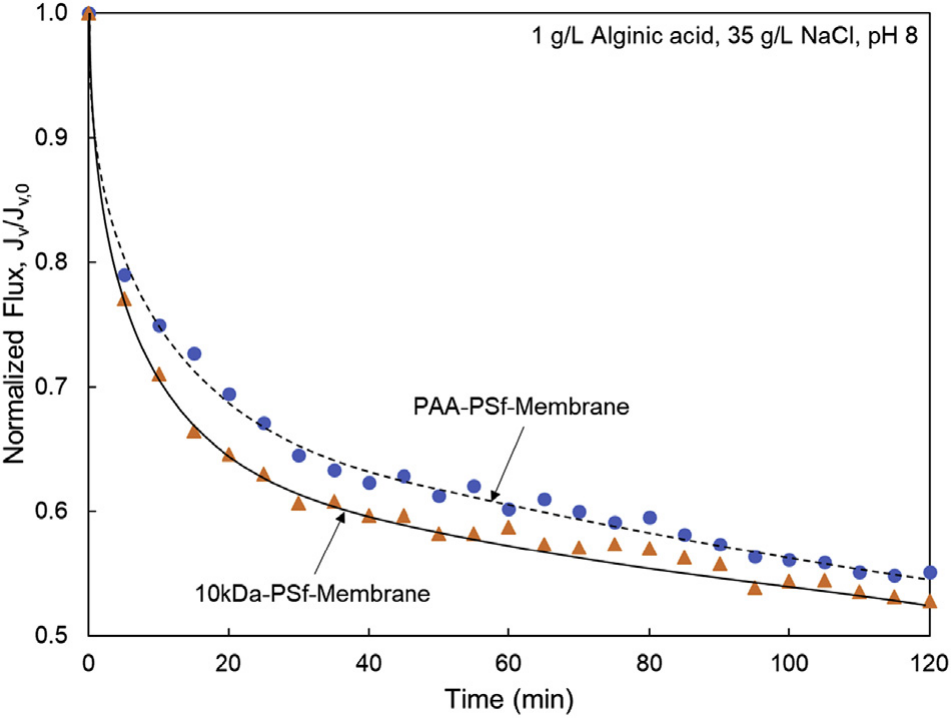
Permeate flux decline during filtration of saline alginic acid solution at pH 8 with (a) PAA-PSf-Membrane (synthesized at [M]_0_ = 20 vol% for 1 h post PSf surface activation with He/O_2_ plasma) and (b) 10kDa-PSf-Membrane.

**Fig. 13:**
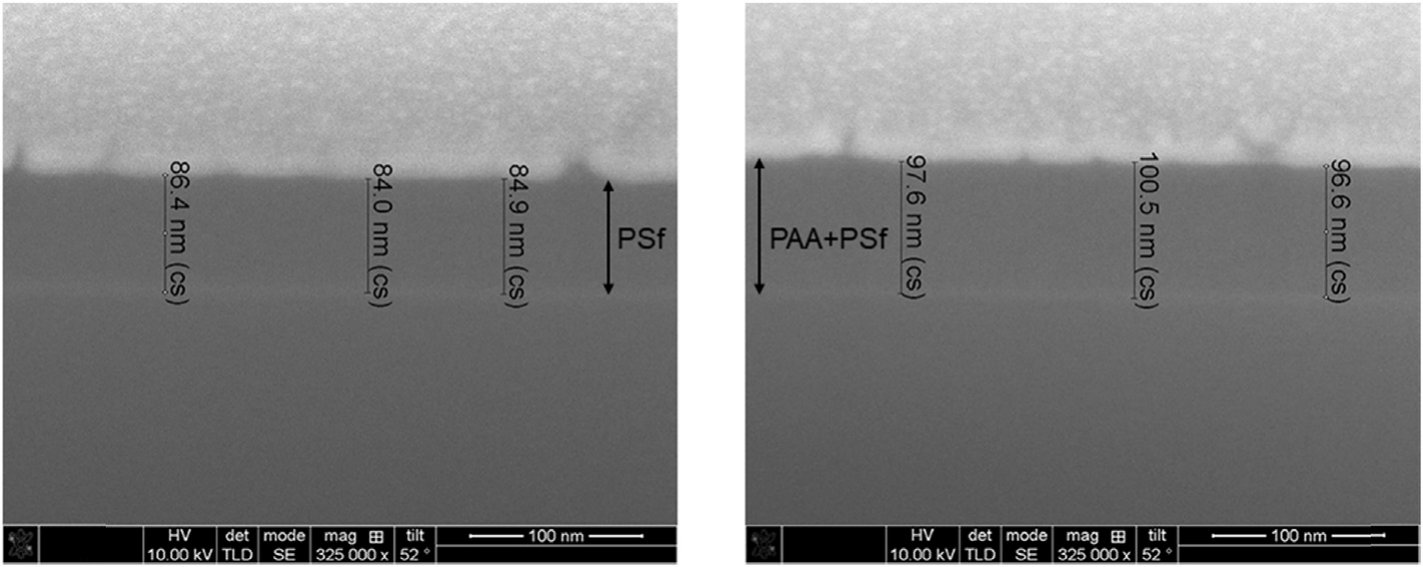
Examples of FIB-SEM images obtained for PSf-Si (left) and PAA-PSf-Si (right) (Note: the tethered PAA layer synthesized at [M]_0_ = 20 vol% for 2 h following surface activation with He/O_2_ APP) used to estimate dry thickness of the PAA layer (by subtracting thickness for the native PSf layer (left) from PAA layer grafted PSf (right)). Note: prior to FIB, a gold layer was deposited onto the samples using a Denton Desk II Sputter Coater (Denton Vacuum, Inc., Moorestown, NJ), followed by coating a thin strip of platinum above the cross-sectioned regions.

Tethered polyacrylic acid layers were synthesized via free-radical graft polymerization at initial monomer concentration ([M]_0_) of 1–20 vol%. The resulting surfaces were characterized with respect to elemental surface composition determined via XPS (Table 1) and surface topography evaluated via AFM analysis (Fig. 1). Contact angles for the different surfaces (Fig. 9, Fig. 10, Table 2, Table 3) were evaluated via the sessile drop method in air and via the captive bubble method in DI water and saline water (35 g/L NaCl)), and the polar and dispersive components of the surface free energies are provided in Table 2. Typical AFM-FS retraction force curves for the native PSf film and PAA layer grafted PSf film surfaces are shown in Fig. 2 and Fig. 4, respectively. The AFM-FS determined chain rupture length and rupture force distribution data for the tethered PAA chains are presented in Fig. 5, Fig. 6, respectively, and the rupture force distribution for the native PSf film in water is shown in Fig. 3. Equilibrium thicknesses for the PAA layers in DI water and in saline water (35 g/L NaCl) are presented in Fig. 7, Fig. 8, respectively, while dry layer thickness data (evaluated via FIB-SEM, Fig. 13) are shown in Fig. 10. Finally, permeate flux decline during filtration of saline alginic acid solution with PAA grafted PSf membrane and native PSf membrane at pH 6 and pH 8 are presented in Fig. 11, Fig. 12, respectively.

**Table 1:** Elemental surface compositions for the native PSf-Si and PAA-PSf-Si^a^.

Surface	Carbon%	Oxygen%	Sulfur%
Native PSf	85.2	11.3	3.5
*Tethered PAA layers synthesized at [M]*_*0*_*:*			
1 vol%	80.9	17.0	2.1
5 vol%	80.4	17.5	2.1
10 vol%	80.1	17.7	2.1
15 vol%	79.1	19.0	1.9
20 vol%	75.4	23.4	1.2

a AA graft polymerization was carried out for a period of 2 h following PSf surface activation by He/O_2_ plasma for a period of 15 s.

**Table 2:** Sessile drop (SD) and captive bubble (CB) contact angles (CA) and surface free energy (SFE) data for the native PSf-Si and PAA-PSf-Si^a^ surfaces.

Surface	SD CA^b^ (°)	CB CA^c^ (°) (air)	CB CA^c^ (°) (octane)	$\gamma_{s}^{p}$^d^ (mJ m^−2^)	$\gamma_{s}^{d}$^d^ (mJ m^−2^)	% Polar SFE component^d^
*Native PSf*	90.0	80.0	118.5	3.5	39.4	8.1
*Tethered PAA layers synthesized at [M]*_*0*_*:*						
1 vol%	49.4	36.6	42.5	38.3	20.8	64.8
5 vol%	49.3	36.6	41.7	38.7	20.4	65.5
10 vol%	47.9	36.8	43.2	38.0	21.1	64.3
15 vol%	46.1	36.4	42.7	38.2	21.1	64.4
20 vol%	44.5	36.9	43.6	37.8	21.3	63.9

a AA graft polymerization was carried out for a period of 2 h following PSf surface activation by He/O_2_ plasma for a period of 15 s.

b Sessile drop contact angles measured with 1 μL DI water drops.

c Captive bubble contact angles measured with 4 μL air and octane bubbles.

d Polar ($\gamma_{s}^{p}$) and dispersive ($\gamma_{s}^{d}$) components of the surface free energy.

**Table 3:** Captive bubble contact angles for PAA-PSf-Si (the tethered PAA layer was synthesized at [M]_0_ = 20 vol% for 2 h post PSf activation with He/O_2_ plasma) in DI water and NaCl solutions.

Solvent	Captive bubble contact angle (°)
DI water (pH 6)	38.4
DI water (pH 8)	29.7
35 g/L NaCl (pH 6)	29.3
35 g/L NaCl (pH 8)	29.1

## 2 Experimental design, materials, and methods

### 2.1 Materials

Helium (He), hydrogen (H_2_) and oxygen (O_2_) gases were used for plasma generation, and nitrogen (N_2_) was utilized for drying and for degassing solvents. Poly (ethyleneimine) (PEI, M_w_ ∼750,000) solution (50 wt% in water), PSf pellets (M_w_ ∼35,000), chloroform and acrylic acid were all utilized in the synthesis of PAA tethered surfaces. Piranha solutions were prepared using sulfuric acid (H_2_SO_4_, 96%) and hydrogen peroxide (H_2_O_2_, 30% in water). Sodium chloride and deionized (DI) water (pH ∼6) were used for preparation of the saline water (35 g/L NaCl). The model foulant [2] for fouling tests was alginic acid sodium salt, and the prepared alginic acid solution pH was adjusted with 50 wt% aqueous sodium hydroxide solution. Finally, membranes with tethered chains were prepared using PSf base membrane of 100 kDa molecular weight cutoff (MWCO).

### 2.2 PSf film preparation

Smooth PSf substrate films were prepared on prime grade silicon wafers by spin-coating a PSf solution using a spin coater. A piranha solution (a 7:3 mixture of concentrated H_2_SO_4_ and 30% aqueous H_2_O_2_) was used to clean the silicon wafers at 90–100 °C for 10 minutes, followed by rinsing with DI water. Silicon wafer samples (∼1 cm × 1 cm) were prepared and cleaned with isopropanol and then DI water, followed by blow drying with N_2_. The wafer surface was first coated with PEI (using ∼0.1 mL of a 0.3 wt% aqueous PEI solution at a spin rate of 2500 rpm for 30 seconds). Subsequently, PSf was coated onto the silicon wafer with PEI coating using ∼0.1 mL of a 1 wt% PSf solution in chloroform that was spin-coated at 2500 rpm for 30 s. The produced PSf-Si substrates were then vacuum oven dried at ∼75 °C prior to their subsequent use.

### 2.3 Plasma surface activation and graft polymerization

An impinging atmospheric pressure plasma (APP) jet [3] was used to activate the PSf substrates using He, He/H_2_ or He/O_2_ plasmas generated at the same He flow rate. All plasmas were generated at 60 W. Graft polymerization of acrylic acid onto the activated surfaces was carried out at monomer concentration of 1–20 vol% over a period of up to 2 h at 70 °C. The DI water rinsed PAA-PSf-Si samples were vacuum dried at 40 °C prior to surface characterization. Tethered PAA layers were synthesized onto a plasma activated base PSf membrane having 100 kDa MW cutoff. Following graft polymerization, the PAA-PSf-Membrane samples were rinsed and stored in DI water prior to ultrafiltration fouling tests.

### 2.4 Surface characterization

Confirmation of the presence of tethered PAA chains on the PSf surface was obtained by X-ray photo electron spectroscopy (XPS). Survey spectra were obtained for both the native PSf-Si and PAA-PSf-Si surfaces at a pass energy of 160 eV. Contact angles for the different surfaces were measured by both the sessile drop (SD) and captive bubble (CB) methods. CB contact angle measurements were for sample immersed in water or saline water at 20–22 °C [4], [5], [6] allowing for ∼30 min equilibration prior to taking measurements [7]. All reported contact angles are averages based on measurements at five different locations on each sample. The surface free energy ($\gamma_{s}$, expressed as the sum of dispersive ($\gamma_{s}^{d}$) and polar ($\gamma_{s}^{p}$) components, $\gamma_{s}=\gamma_{s}^{d}+\gamma_{s}^{p}$) was determined from the average air and *n*-octane CB contact angles (*θ*) following the method described in Refs. [8], [9], which required the dispersive, polar, and total surface tension of liquids (i.e., water and *n*-octane) reported in Ref. [6].

Atomic force microscopy (AFM) (Bruker Dimension Icon Scanning Probe Microscope with a NanoScope V Controller, Bruker, Santa Barbara, CA) was utilized to obtain surface feature height data in PeakForce Tapping mode, under air, DI water and saline water (35 g/L NaCl), using ScanAsyst-Air and ScanAsyst-Fluid + probes (Bruker AFM Probes, Camarillo, CA). Cantilever deflection sensitivity and spring constant were determined following the approaches specified in Refs. [10], [11], respectively. All AFM scans were obtained for areas of 1 μm × 1 μm at 512 × 512-pixel resolution and 0.5–0.8 Hz scan rates using a relatively small loading force (i.e., ∼500 pN).

AFM force spectroscopy (AFM-FS; [12], [13], [14]) was accomplished using a Bruker MultiMode 8-HR Scanning Probe Microscope with a PicoForce Spectroscopy Control Module (Bruker, Santa Barbara, CA) under DI water and 35 g/L NaCl solution. A silicon nitride probe of a nominal tip radius of 20 nm (MLCT-D, Bruker AFM Probes, Camarillo, CA) was used, and force measurements were taken in contact mode at 1 μm ramp size and 500 nm/s tip velocity. The maximum surface applied force prior to tip retraction (i.e., trigger force) was 1 nN, and the approach and retraction force-distance profiles were obtained from 200 randomly selected locations for each sample. The rupture force and rupture length distributions as well as the equilibrium thickness [1] were determined from the retraction force profiles.

Sectioned substrate images were obtained via Focused Ion Beam (FIB) – Scanning Electron Microscopy (SEM) (Nova 600 NanoLab DualBeam^TM^-SEM/FIB; FEI company, Hilsboro, OR) [15]. The dry PAA layer thickness was calculated as the difference between the film thickness above the silicon wafer substrate before and after graft polymerization (an example of the thickness analysis is illustrated in Fig. 13).

### 2.5 Ultrafiltration fouling tests

Membrane fouling tests for the PSf (10 kDa MWCO) and PAA-PSf membranes were performed in a dead-end filtration mode (at 20 °C) using a small (50 mL) stirred cell system (MilliporeSigma, Burlington, MA) of 13.4 cm^2^ membrane filtration area. Fouling challenge tests were conducted with a 1 g/L alginic acid solution at pH 8 and at pH 6 in high salinity water (35 g/L NaCl). All filtration tests were carried out at 22 L m^−2^ h^−1^ initial permeate flux which is well within range of permeate flux for UF seawater pretreatment [16].
