# Supplementary material for: Surface characterization data for tethered polyacrylic acid layers synthesized on polysulfone surfaces
Source: Data Brief. 2019 Mar 7;23:103747. doi: 10.1016/j.dib.2019.103747 (PMC6660637; doi:10.1016/j.dib.2019.103747)
Supplement: Supplementary file 1 — Multimedia component 1 [file mmc1.docx]

Declarations of Interest: none
